# Stimuli-Responsive Cationic Hydrogels in Drug Delivery Applications

**DOI:** 10.3390/gels4010013

**Published:** 2018-02-01

**Authors:** G. Roshan Deen, Xian Jun Loh

**Affiliations:** 1 Soft Materials Laboratory, Natural Sciences and Science Education AG, National Institute of Education, Nanyang Technological University, 1-Nanyang Walk, Singapore 637616, Singapore; 2 Institute of Materials Research and Engineering, 2-Fusionopolis Way, Singapore 138634, Singapore; lohxj@imre.a-star.edu.sg

**Keywords:** cationic polymers, hydrogels, stimuli-responsive, heterocyclic, swelling of gels, drug-delivery systems

## Abstract

Stimuli-responsive, smart, intelligent, or environmentally sensitive polymers respond to changes in external stimuli such as pH, temperature, ionic strength, surfactants, pressure, light, biomolecules, and magnetic field. These materials are developed in various network architectures such as block copolymers, crosslinked hydrogels, nanogels, inter-penetrating networks, and dendrimers. Stimuli-responsive cationic polymers and hydrogels are an interesting class of “smart” materials that respond reversibly to changes in external pH. These materials have the ability to swell extensively in solutions of acidic pH and de-swell or shrink in solutions of alkaline pH. This reversible swelling-shrinking property brought about by changes in external pH conditions makes these materials useful in a wide range of applications such as drug delivery systems and chemical sensors. This article focuses mainly on the properties of these interesting materials and their applications in drug delivery systems.

## 1. Introduction

**Figure 1:**
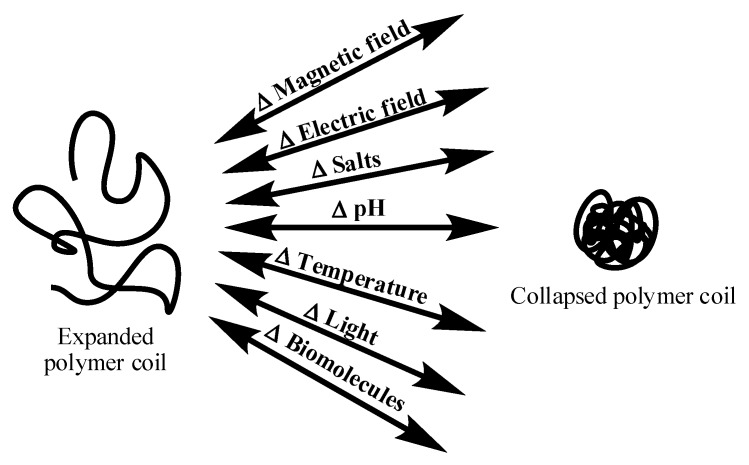
Stimuli-response behavior of polymer chains in response to various external stimuli.

Polymers that respond to changes in external stimuli are called stimuli-responsive, smart, intelligent, or environmentally sensitive polymers. These materials can be in the form of linear polymers (homo polymers, copolymers), crosslinked polymers in the form of hydrogels, inter-penetrating networks, and micro/nanogels. The external stimuli can be either physical, chemical, or biochemical in nature, in the form of pH, temperature, salts, surfactants, light, pressure, biomolecules, and magnetic field [1,2,3,4,5]. The responses to these external stimuli occur in the form of conformational, optical, and chemical changes. The different types of stimuli that bring about these changes are illustrated in Figure 1. These changes are accompanied by variation in the physical properties of the polymer. On a macroscopic level, the changes are apparent as phase separation from aqueous solution (for linear polymers and linear copolymers) or volume changes (for crosslinked systems) [5,6,7,8,9,10]. The temperature at which such transitions occur is termed the lower critical solution temperature (LCST) or volume-phase transition temperature (VPT) [11]. This article focuses specifically on cationic polymers in the form of hydrogels and their applications in drug delivery applications. 

**Figure 2:**
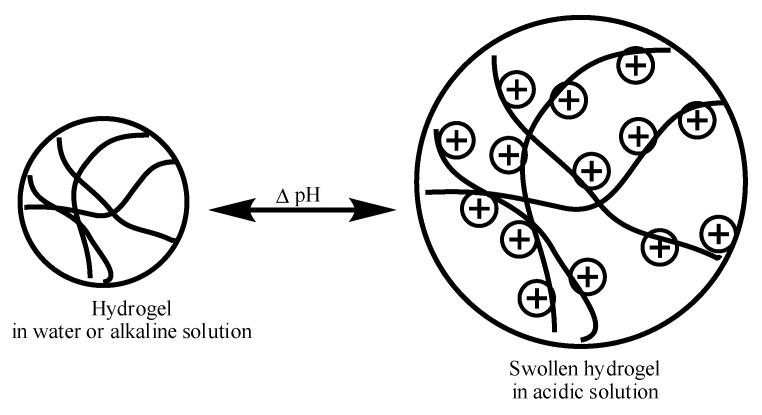
Swelling of hydrogel in acidic solution due to the formation of fixed charges on the polymer network.

**Figure 3:**
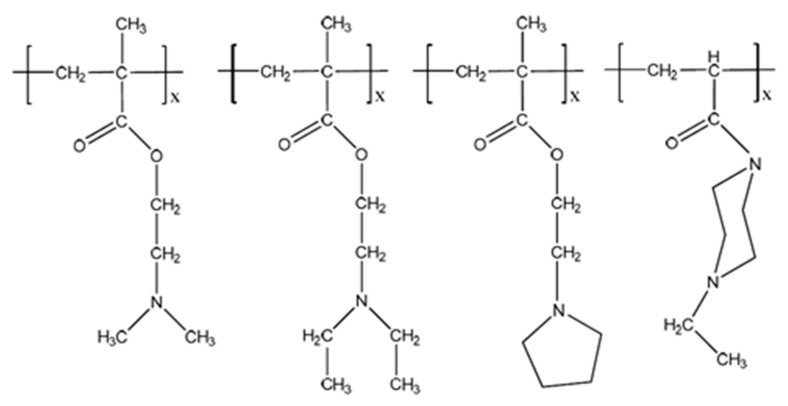
Chemical structure of cationic polymers (1) poly(dimethylaminoethyl methacrylate) (PDMAEMA); (2) poly(diethylaminoethyl methacrylate) (PDEAEMA); (3) poly(ethyl pyrrolidine methacrylate) (PEP); (4) poly(ethyl piperazine acrylate) (PAcrNEP).

Hydrogels that contain polycations and are sensitive to external pH changes are called cationic hydrogels. The tertiary amine functional groups present in these hydrogels are protonated below their dissociation constant (*pK*_a_) in acidic solutions, causing the hydrogels to swell extensively. This pH-dependent swelling is shown in Figure 2 [12,13,14,15,16]. A few examples of cationic hydrogels are based on poly(*N*,*N*-dialkylaminoethyl methacrylate), poly(lysine), chitosan, poly(amido-amine), etc. The chemical structures of some pH-sensitive synthetic cationic polymers such as poly(dimethylaminoethyl methacrylate) (PDMAEMA), poly(diethylaminoethyl methacrylate) (PDEAEMA), poly(ethyl pyrrolidine methacrylate) (PEP), and poly(ethyl piperazine acrylate) (PAcrNEP) are shown in Figure 3. Hydrogels based on these chemical moieties swell extensively in solutions with a low pH due to the protonation of tertiary amine functional groups, which leads to the formation of fixed electric charges on the macromolecule. The degree of swelling of cationic hydrogel depends largely on the *pK*_a_ of the ionic group, concentration of the monomer, crosslinking ratio, pH, and ionic strength of the external medium [17,18]. 

In recent years, cationic hydrogels that exhibit response to more than one stimuli, such as pH and temperature, have attracted much research attention in biomedical applications. These polymeric materials are obtained from new synthetic monomers containing tertiary amine functional groups [17,18,19]. This has been achieved by the following chemical synthesis approaches: (i) the copolymerization of monomers with desired functional groups [20,21,22,23,24,25], (ii) the copolymerization or combination of thermo-sensitive polymers with polyelectrolytes [26,27], (iii) the formation of inter-penetrating networks through combination of various stimuli-sensitive polymers [28,29], and (iv) the chemical synthesis and polymerization of new functional monomers [30,31].

Cationic polymers and hydrogels are an interesting class due to their pH sensitivity and due to their ability to complex with other systems with anionic character [32]. As a result of these characteristics, cationic polymers and hydrogels have been widely evaluated as alternative vectors to viruses in gene delivery and therapy [33,34]. The nature of cationic groups and their influence on transfection efficiency and biodegradability has been studied for cationic polymers based on poly(phosphoramides) [33,34,35]. 

## 2. Swelling Behavior of Cationic Hydrogels

The most favorable property of hydrogels is their ability to swell when placed in contact with a thermodynamically compatible solvent. Solvent molecules penetrate the glassy surface of the hydrogel and slowly diffuse into the network. The physicochemical models that describe the swelling of cationic hydrogels are usually based on the free energy change that takes place due to the following factors [34,35,36,37]: (i) the osmotic pressure of counterions within the gel (Donnan theory); (ii) the mixing of polymer with solvent (Flory-Huggins theory); (iii) the stretching of polymer chains (Flory-Rehner theory). 

The total free energy change of a hydrogel at equilibrium swelling is given by the Gibbs free energy equation [38]:

(1) $$\Delta G_{Total}=\Delta G_{Mixing}+\Delta G_{Elastic}$$

where Δ*G_Total_* is the change of total free energy in hydrogel, Δ*G_mixing_* is the free energy due to the mixing of solvent molecules with polymer chains, and Δ*G_elastic_* is the free energy of the elastic retractive force of the hydrogel. 

In the case of ionic hydrogels, the theoretical treatment of equilibrium swelling is more complicated, as swelling depends to a large extent on the degree of ionization of functional groups, as well as the ionic strength of the external solution. Therefore, the ionic groups impart an additional free energy change (Δ*G_ionic_*) to the total free energy of the hydrogel. The total free energy change of the ionic hydrogel is given as:

(2) $$\Delta G_{Total}=\Delta G_{Mixing}+\Delta G_{Elastic}+\Delta G_{ionic}$$

The ionic gel is subjected to a swelling pressure, *π*, which comprises three components similar to the total free energy:

(3) $$\pi_{Total}=\pi_{mixing}+\pi_{elastic}+\pi_{ionic}$$

where *π_mix_* is the osmotic pressure due to the mixing of solvent with polymer, *π_elastic_* is the osmotic pressure due to the elastic force of the gel, and *π_ionic_* is the osmotic pressure due to the ionic contribution. The equilibrium swelling is obtained when *π_Total_* is set equal to zero. 

The osmotic pressure due to mixing, *π_mix_*, is given by the Flory-Huggins theory [36]:

(4) $$\pi_{mixing}=-\frac{RT}{V_{1}}\left[\ln\;(1-\upsilon )+\upsilon +\chi \upsilon^{2}\right]$$

where *υ* is the polymer volume fraction, *V*_1_ is the molar volume of solvent, and *χ* is the Flory-Huggins interaction parameter. 

The osmotic pressure due to elastic or configurational contribution (*π_elastic_*) is obtained from Δ*G_elastic_*, during the swelling of hydrogels. Under isotropic swelling, this is obtained by differentiating Δ*G_elastic_* with respect to volume and expanding the inverse Langevin function in a power series, as given by the following expression [39]:

(5) $$\begin{array}{l} \pi_{elastic}=-v_{o}RT\left[{\left(\frac{\upsilon }{\upsilon_{0}}\right)}^{1/3}-\frac{1}{2}\left(\frac{\upsilon }{\upsilon_{0}}\right)\right]-v_{0}RT\\ \left[\frac{3}{5}{\left(\frac{\upsilon_{0}}{\upsilon }\right)}^{1/3}\times \frac{1}{n}=\frac{99}{175}\left(\frac{\upsilon_{0}}{\upsilon }\right)\frac{1}{n^{2}}+\frac{513}{875}{\left(\frac{\upsilon_{0}}{\upsilon }\right)}^{5/3}\frac{1}{n^{3}}+\ldots \right] \end{array}$$

where *v*_0_ = *υ*_0_ *v*_d_ is the concentration of polymer chains when the gel is formed.

The ionic contribution to the osmotic pressure (*π_ion_*) arises due to the difference between the osmotic pressure of mobile ions in the gel and in the external solution. This is given by [39]:

(6) $$\pi_{ionic}=RT\left[\Phi \sum_{i}\bar{C_{i}}-\varphi \sum_{i}C_{i}\right]$$

where *C*_i_ and $\bar{C_{i}}$ are the concentrations of mobile ions in the external solution and gel; *φ* and Φ are the corresponding osmotic coefficients, respectively. This implies that charged or ionizable groups present in gels play an important role in swelling behavior. The following equation describes the swelling of cationic hydrogels in the presence of a solvent and their abovementioned dependencies [37,38,39]. Using this expression, the network structure of cationic gels can be characterized.

(7) $$\begin{array}{l} \frac{V_{1}}{4I}\left(\frac{v_{2,s}^{2}}{\bar{v}}\right){\left(\frac{K_{b}}{10^{\mathrm{pH}-14}-K_{a}}\right)}^{2}=\left[\ln\left(1-v_{2,s}\right)+v_{2,s}+\chi v_{2,s}^{2}\right]+\\ \left(\frac{V_{1}}{v{\bar{M}}_{c}}\right)\times \left(1-\frac{2{\bar{M}}_{c}}{{\bar{M}}_{n}}\right)v_{2,r}\left\lfloor {\left(\frac{v_{2,s}}{v_{2,r}}\right)}^{1/3}-\left(\frac{v_{2,s}}{2v_{2,r}}\right)\right\rfloor \end{array}$$

where *I* is the ionic strength, *K*_a_ is the acid dissociation constant, *K*_b_ is the base dissociation constant, *M_c_* is the molecular weight between crosslinks, *M_n_* is the molecular weight of polymer chains without crosslinks, *V*_1_ is the molar volume of water, *v*_2_, *r* is the volume fraction of the polymer in the relaxed state, *v*_2_,*s* is the volume fraction of the polymer in the swollen state, and *χ* is the polymer-solvent interaction parameter.

## 3. Applications of Cationic Hydrogels

Cationic hydrogels have found several applications in the biomedical industry such as targeted drug delivery, gene delivery, and tissue engineering.

### 3.1. Drug Delivery Systems

The process of administering a pharmaceutical compound to a targeted organ to achieve a therapeutic effect in humans or animals is the principle of targeted drug delivery systems. Due to the variation of pH (between 2 in the stomach to neutral in the small intestine) along the gastrointestinal tract (GI), it is still the most sought after route for drug delivery [40]. It is also the most complex route and therefore various approaches are required for the effective delivery of drugs. A few of the obstacles that need to be overcome for efficient drug delivery include the degradation of drugs and the carrier (gels) by enzymes, rapid removal of the carrier from the body, non-specific toxicity of carrier, etc. In this regard, stimuli-responsive systems allow the advantage of delivering the right amount of drug at the right time in response to changes in external stimuli. Cationic gels, for example, can expand as a result of the ionization of functional moieties present along the macromolecular chain in an acidic condition, thus promoting drug diffusion and release in the stomach [40]. 

Several cationic hydrogels based on heterocyclic compounds, such as morpholine and pyrolidinone, have been widely studied by San Román and co-workers [27,28,30]. The same group also developed polymers in which an anti-aggregant drug called Triflusal was covalently attached (polymeric prodrugs) [41]. A novel poly(vinylpyrrolidone-co-dimethylmaleic anhydride) (PVD) carried drug was synthesized by Kamada and co-workers [42]. The incorporated pH-sensitive vinylpyrrolidone cationic monomers allowed the conjugation of the drug Adriamycin at pH 8.5, with gradual release at pH 6 to 7. The PVD-Adriamycin conjugate has shown anti-tumor activity against Sarcoma-180 solid tumor in mice. 

An injectable hydrogel based on vinylpyrrolidine, NIPAM, and acrylamide was reported by You and co-workers [43]. This hydrogel swelled in a solution of pH 6.5 at 25 °C and shrunk in a solution of pH 8.5 at 37 °C. The combined pH and temperature sensitivity facilitated the extended delivery of an opioid receptor antagonist, naltrexone, over a period of 28 days. Cationic graft copolymers in the form of nanoparticles based on PDMAEMA and polycaprolactone (PCL) were prepared by Gua and co-workers [44] for the encapsulation of paclitaxel and hydrophilic biomolecules. A faster release of the drug was achieved in solutions with a low pH.

Cationic polymers have also played a role in the formulation of sustained release matrix tablets. Matrix tablets were prepared from a combination of hydrophobic ethyl cellulose and hydrophilic sodium carboxymethyl cellulose polymers. The in vitro release of losartan potassium (drug for hypertension treatment) from the matrix tablet was studied. The results showed that the formulation produced sustained drug release over a period of 12 h. Cationic polymers in the form of biodegradable micelles have also been reported as drug carriers [45,46,47]. The polymer micelles were based on PDMAEMA-PCL-PDMAEMA triblocks. Successful delivery of paclitaxel into tumor cells was achieved, thus optimizing the treatment of tumor cells using cationic polymer micelles. Cationic polymers containing disulfide links prepared through a Michael addition reaction was found to conjugate doxorubicine. The polymer-drug conjugate displayed good stability in physiological pH conditions [48]. 

Amphiphilic cellulose cationic micelles were prepared by Song and co-workers [49]. This polymer self-assembled into spherical micelles in water which was used as a carrier for the lipophilic drug, prednisolone. Micelles based on PNIPAM, DMAM, and poly (lactic acid) (PLA) were prepared by Akimoto and co-workers [50]. These micelles were able to diffuse into the cells above the LCST of the polymer, due to increased interaction between the solvated micelles and cells. 

ABA type triblock cationic copolymers based on PDMAEMA (block A) and PVCL (block B) were prepared by San Miguel and co-workers [51]. The formation of pH- and temperature-responsive micelles as well as in vitro sustained drug release were demonstrated in this study. 

### 3.2. Gene Delivery Systems

The process of administering a gene (DNA) for correcting defective genes to achieve the treatment of many genetic diseases is the principle of gene delivery/gene therapy systems. The delivery of the appropriate therapeutic gene into the cell to replace or regulate the defective gene is a vital step in gene delivery. The gene is transported in gene delivery carriers called gene carriers or vectors or vehicles. DNA is a negatively charged hydrophilic molecule that has a large size at physiological conditions and is therefore very difficult to incorporate into cells. Liposomes and polycations are two important classes of non-viral chemical gene delivery methods to condense DNA that can be transported into cell to replace the defective gene [52]. 

This gene delivery process using a cationic polymer or hydrogel is also called transfection. This process involves four main steps:

(1) Complexation for DNA with the cationic polymer/hydrogel. The DNA-cationic polymer/hydrogel is termed as a polyplex.
(2) Addition of the polyplex to the cell containing the defective DNA for a certain period of time.
(3) Release of DNA into the cytoplasm and removal of cationic polymer/hydrogel.
(4) Transfer of DNA into nucleus. This step involves incubation for a period of time until the desired results are obtained.

Cationic polymers can either complex with and condense DNA into small particles, or combine with DNA through conjugation, in which the covalent bond will be cleaved in order to release the DNA. To note, a major demerit of many polymer-based non-viral vectors, such as poly(ethyleneimine), PDMAEMA, and dendrimers based on poly(amido-amine), is that they show significant cytotoxicity and lower transfection efficiency [45]. Interestingly, polyplexes based on biodegradable cationic polymers show lower cytotoxicity and improved transfection efficiencies. Such polymers include poly(4-hydroxy-l-proline ester), poly[α-(4-aminobutyl)-l-glycolic acid] (PAGA), cationic polyphosphazenes, and linear or branched poly(amino ester)s [53,54,55]. 

Cationic polymers based on poly(amido amine)s show improved stability against hydrolysis compared to poly(amino ester)s, as the amide group is less sensitive to hydrolysis than the ester group. Ferruti and co-workers [56,57,58,59,60] synthesized and studied the properties and application of a wide variety of poly(amido amine)s. For these polymers, the endosomal escape of the polyplexes was attributed to the protonation of the tertiary amino groups, which induces a conformation change. 

**Figure 4:**
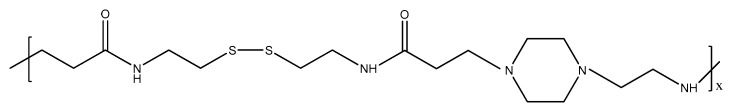
Chemical structure of poly(amido-amine) with disulfide linkage along the polymer backbone.

A few major barriers for non-virial gene delivery are: (i) the inefficient endosomal escape of the polyplexes to avoid the lysosome degradation pathway, and (ii) the unpacking of DNA from the polyplexes to allow transcription [61]. Lin and co-workers [62,63] reported the synthesis of novel poly(amido-amine) linear and copolymers containing disulfide linkages along the polymer backbone (Figure 4). 

These polymers were biodegradable and showed improved biophysical properties. These polymers effectively condensed DNA into polyplexes with a size of less than 150 nm with a positive surface charge. These novel polymers containing disulfide linkages also facilitated polyplex unpacking, leading to enhanced gene expressions and lower levels of cytotoxicity. Hoffmann and co-workers [64] designed and functionalized a new monomer containing a disulfide linkage as a pendant group viz. pyridyl disulfate acrylate (PDSA). This polymer allowed efficient conjugation through disulfide linkages for effective endosomal translocation of therapeutics. 

**Figure 5:**
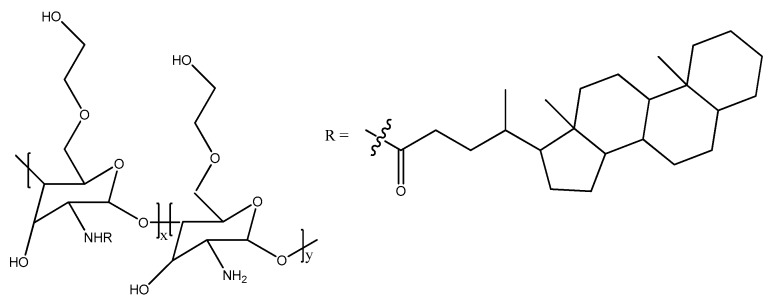
Chemical structure of glycol chitosan modified with cholanic acid.

Hydrophobically modified cationic polymers with small or bulky lipids, such as cholesterol, have facilitated the translocation of DNA/s-RNA complexes through the cell membrane [65]. A biodegradable non-toxic poly[α-(4-aminobutyl)-l-glycolic acid] (PAGA) used as gene carrier was reported by Lim and co-workers [66]. The polymer condensed with DNA and exhibited fast degradation. The transfection efficiency of this polymer with DNA was found to be higher than the poly(l-lysine) analogues. Forrest and co-workers [67] showed that an increase in the hydrophobicity of acylated branched poly(ethylene amine) caused a fourfold uptake of polyplexes and better transfection. Among poly-l-lysine, the cholesterol-modified analogues showed improved transfection. Further, it was also shown that the incorporation of a hydrophobic cholanic acid moiety in glycol chitosan (Figure 5) was essential for the formation of polyplexes of hydrophobic p-DNA [66,67]. 

### 3.3. Tissue Engineering

Natural and synthetic cationic polymers in particular porous hydrogels have been used as scaffolds to engineer various forms of new tissues. The role of most polymer scaffolds is to provide a modified surface of suitable porosity for seeded cell adhesion and interaction, very much like the extracellular matrix. The volume of tissue developed depends on the crosslinking density and the pore size of the polymer scaffolds [68]. Biocompatible and biodegradable polymers, such as cationic chitosan, poly-l-lysine, poly(ethylene imine) (PEI), etc., have been widely used for this purpose. Various PEI polymers [69,70] have been used to fabricate scaffolds for cultivating bovine chondrocytes and normal human fibroblast cells.

The porous scaffolds of chitosan reinforced with calcium phosphate for improved osteo-retention have been used in bone tissue engineering. This reinforced cationic polymer scaffold was used to regenerate new bone tissue of increased fracture and fatigue resistance [71]. Fiber mesh scaffolds and microspheres based on chitosan have also been studied for bone generation, cell adhesion, and viability [72]. Using chitosan and chitin scaffolds grafted with poly(l-lysine) (PLL), the efficient generation of cartilaginous components was achieved, thus showing the potential application of cationic polymers in articular cartilage engineering. 

### 3.4. Nanoparticles and Microparticles Based on Cationic Polymers

Polymeric nanoparticles and microparticles are of special interest because of their properties such as nontoxicity, biocompatibilty, and stimuli-sensitivity. Nano/microparticles based on cationic polymers may enhance the cellular uptake and endosomal escape of the particles [73]. These materials have been synthesized by methods such as solvent evaporation [74], spray drying [75], emulsification [76], ionotropic gelation [77], and controlled polymerization methods such as atom transfer radical polymerization (ATRP) [78]. Nano or microshperes of chitosan have been widely used for the delivery of insulin [79], heparin [80], cyclosporin A [81], and a variety of proteins [82]. An important application of cationic polymers in the form of nanoparticles is in the deliverly of hydrophobic drugs. Almost one-thrid of newly deiscovered drug molecules are sparingly soluble in water [83]. Cationic copolymers based on 5-*Z*-amino-δ-valerolactone and ε-caprolactone assemble into nanoaggregates at concentrations above 0.5 mg mL^−1^, thereby increasing the water solubility of hydrophobic drugs by about 100–1000 times [84]. 

### 3.5. Multilayer Films or Coatings

**Figure 6:**
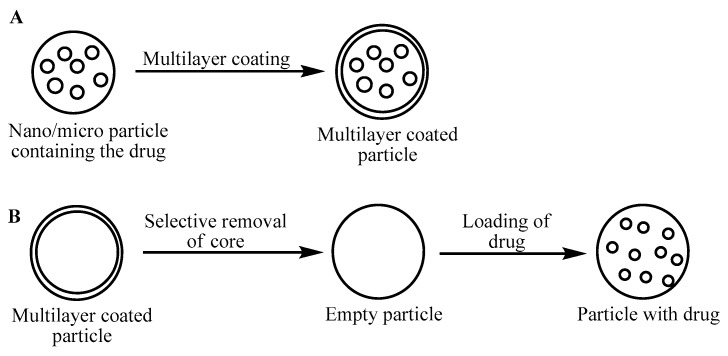
Methods to obtain multilayer polymer films of drug delivery systems. (**A**) Multilayer coating on particle with encapsualted drug; (**B**) sacrificial removal of core followed by drug loading.

Multilayer films of polymers are usually obained by the layer-by-layer method, usually from oppositely charged polyelectrolytes, neutral polymers, cationic dendrimers [85], or polycations. The films may be deposited onto nano/microspheres, forming the desired capsules for drug encapsulation [86]. A schematic represenation of the various methods used to obtain coated multilayer films for drug delivery is shown in Figure 6. The drug can also be introduced into the nano/microshperes before coating them with multilayer film, or the core of the capsule can be selectively removed before encapsulating the drug. In this case, the capsules serve as sacrifical templates [87]. 

The main disadvantage of hollow capsules is the low efficiency of drug loading due to the adsorption of the drug on the walls of the capsule rather than in the core. Various cationic polymers, such as chitosan, protamine, poly(4-vinyl pyridine), poly(diallyl dimethylammonium chloride), poly(l-arginine), poly(dimethyl aminoethyl methacrylate) [88,89,90,91], etc., have been used in the preparation of multilayer films. The number of layers or thickness of the capsules is related to the permeability and the release of the active substances. It has been found that the permeability decreases with an increase in the thickness of the layers [91]. The rate of release of a drug can be greatly controlled by coating the drug directly with a multilayer film. The release rate of furosemide microcrystals coated with cationic polymers with a thickness of 150 nm was found to reduce the rate up to 300 times compared to the uncoated drug [92]. 

## 4. Conclusions

In summary, stimuli-responsive cationic polymers show interesting properties in response to changes in the external pH of the medium. In this paper, a brief introduction to cationic polymers/hydrogels in terms of design, theory of swelling, and recent biomedical applications such as drug and gene delivery and tissue engineering has been compiled. Based on the variety of cationic polymers and their interesting properties, these materials show promise and will have a definite impact on the formation of polymer-based biomaterials and chemical sensors.
